# Pitfall of the Strongest Cells in Static Random Access Memory Physical Unclonable Functions

**DOI:** 10.3390/s18061776

**Published:** 2018-06-01

**Authors:** Mingyang Gong, Hailong Liu, Run Min, Zhenglin Liu

**Affiliations:** School of Optical and Electronic Information, Huazhong University of Science and Technology, Wuhan 430074, China; d201677550@hust.edu.cn (M.G.); D201577539@hust.edu.cn (H.L.); minrun@hust.edu.cn (R.M.)

**Keywords:** PUFs, SRAM, fuzzy extractor, repetition code, clone, ERFE

## Abstract

Static Random Access Memory (SRAM) Physical Unclonable Functions (PUFs) are some of the most popular PUFs that provide a highly-secured solution for secret key storage. Given that PUF responses are noisy, the key reconstruction must use error correcting code (ECC) to reduce the noise. Repetition code is widely used in resource constrained systems as it is concise and lightweight, however, research has shown that repetition codes can lead to information leakage. In this paper we found that the strongest cell distribution in a SRAM array may leak information of the responses of SRAM PUF when the repetition code is directly applied. Experimentally, on an ASIC platform with the HHGRACE 0.13 μm process, we recovered 8.3% of the measured response using the strongest cells revealed by the helper data, and we finally obtained a clone response 79% similar to weak response using the public helper data. We therefore propose Error Resistant Fuzzy Extractor (ERFE), a 4-bit error tolerant fuzzy extractor, that extracts the value of the sum of the responses as a unique key and reduces the failure rate to 1.8 × 10^−8^ with 256 bit entropy.

## 1. Introduction

Electronic devices have never been used so widely in our lives as now. With the dramatic development of electronic payments, tag applications, and Internet of Things (IoTs), the issue of equipment security issue has become increasingly severe, particularly in the generation, distribution, storage, and destruction of secret keys [1,2]. The security of secret keys can be solved using Physical Unclonable Functions (PUFs) [3,4].

Pappu [5] proposed the concept of PUFs and presented the first method for PUF hardware design. Since then, many different types of PUFs have been proposed such as arbiter PUF [6], Ring Oscillator (RO) PUF [7], Static Random Access Memory (SRAM) PUF [8], butterfly PUF [9], and double-data-rate SDRAM Type 3 (DDR3) PUF [10]. Among these PUFs, SRAM PUF is widely used because of the following advantages [11,12]: (1) SRAM is a standard component existing on different process nodes, and (2) many chips have built-in SRAM arrays as caches and temporary data storage units that can be used as PUFs to generate Challenge-Response pairs (CRPs) without requiring additional design and area overhead compared to other types of PUFs.

The responses are noisy; they cannot be directly used as cryptographic keys. A fuzzy extractor is generally required to extract the correct key from the responses [13]. Bösch et al. proposed a cost friendly fuzzy extractor hardware scheme by concatenating two error correction codes [14]. Repetition code is simple, efficient, and requires only a small number of logic cells, being generally used as outer code to decrease the error rate of measured responses. Another error correction code like BCH code, Reed-Muller code (RM), and Golay code is used as inner code to eliminate the remaining errors [14,15,16].

Rührmair et al. classified PUFs into strong PUFs and weak PUFs according to the relationship between the size of the challenge-response pairs (CRPs) [17]. Strong PUFs have a huge challenge space that exponentially related to the size of CRPs. Gassend et al. successfully attacked arbiter PUF, which is a type of strong PUF using standard machine learning (ML) methods, such as the support vector machine (SVM) and perceptrons method, after collecting a number of CRPs [18]. They used the collected CRPs of the PUF to train the ML algorithm, then predicted the key based on the responses. The prediction accuracy can be significantly improved when the number of training CRPs is sufficiently large.

Koeberl et al. studied the entropy characteristics of PUFs [15]. They found that using repetition codes would reduce the leftover entropy to zero when the entropy of the PUF responses was less than 66%. Since a small decrease in PUF entropy can result in zero leftover entropy, high entropy is required in PUF design and application. The authors theoretically showed the risk of low entropy, but they did not provide scenarios with low entropy or the factors that may decrease the entropy. 

Delvaux et al. attacked the arbiter PUF using side channel modeling [19] and found that the physical information leaked in the side channel attack could be used to train the ML algorithm, resulting in higher ML prediction accuracy. 

Helfmeier et al. proposed a method of cloning the responses of a SRAM using a focused ion beam (FIB) [20]. By learning the characteristic parameters of the SRAM array, they trained the corresponding transistors on the target SRAM using FIB, so that the target SRAM had the same responses as the cloned SRAM. This cloning method requires the use of expensive and complex FIB devices, and hence complicating the cloning of the responses of a large SRAM array. 

Xiao et al. found that strong cells exist in SRAM arrays. Cells having the same response during every power-on are strong cells and they proposed a scheme of extracting strong cells as a PUF key [21]. This PUF uses a strong cell with low bit error rate, significantly decreasing the overhead of the error correcting code.

In this paper, we analyze the distribution of the SRAM power-on values. We found that SRAM PUFs have weak responses when a repetition code is directly used, and the distribution of the strongest cells in the SRAM array further reduces the leftover entropy of the PUF. Using the helper data generated by the repetition code, combined with the distribution of the strongest cells, we recovered 8.3% of the data from the responses of the SRAM PUF. The information leakage of the PUF caused by weak responses and strongest cell distribution led to zero leftover entropy [15]. In addition, we propose Error Resistant Fuzzy Extractor (ERFE), a lightweight extractor that does not leak the information of the strongest cells. Our contributions are as follows:

(1) We first propose a method to recover the response using the helper data from a SRAM PUF with the distribution of the strongest cells. Many previous studies [15,22,23] theoretically hypothesized that the helper data would reduce the entropy and then lead to the risk of leakage of PUF responses. Our research experimentally proves this risk.
(2) Our research reveals that the SRAM PUF based on repetition codes would generate a weak responses. The helper data generated using the weak responses reveal information about the PUFs’ responses.
(3) We also propose ERFE with 4-bit error tolerant ability, which extracts cells’ sum value as the key.

The structure of this paper is as follows: in Section 2, we review the structure and characteristics of SRAM PUFs. In Section 3, we study and analyze the weakness of SRAM PUFs based on repetition code and use the helper data generated by the weak responses to generate a clone of the power-on value of the SRAM. Section 4 describes the ERFE. The experimental results are discussed in Section 5. We conclude this paper in Section 6.

## 2. Related Work

### 2.1. Structure of an SRAM Array

**Figure 1:**
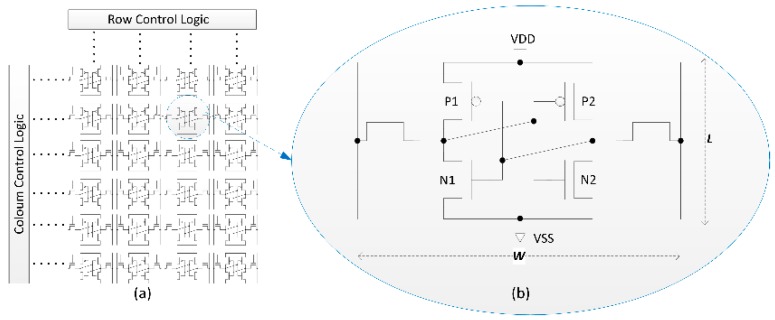
Structure of SRAM array. (**a**) SRAM array and (**b**) 6T SRAM cell with width *W* and length *L*.

A SRAM PUF uses the power-on value of the SRAM array as responses. The SRAM array consists of many 6T SRAM cells, each cell is a regular rectangle in circuit layout with width *W* and length *L*, as shown in Figure 1. P1 and P2 are positive channel Metal Oxide Semiconductor (PMOS), and N1 and N2 are negative channel Metal Oxide Semiconductor (NMOS). P1 and N1, and P2 and N2 make up two inverters. When power is off, both inverters output zero. When power-up, N1 and N2 are off, P1 and P2 are on and try to push the outputs of both inverters to power voltage (VDD). When the output voltage on each side reaches the NMOS turn-on voltage, each NMOS is turned on and tries to pull the output voltage back to zero. In an ideal situation, the inverters on both sides and the voltage rising speed of both sides are the same, so the final output of the cell is an indefinite state. Due to manufacturing process variation, the inverters on both sides are not identical, and the currents flowing through the two inverters are not the same. The consequence is that the PMOS output voltage on one side increases faster than on the other and reaches the NMOS turn-on voltage first. The output voltage of the other inverter will be pulled back to zero, driving the cell value to zero or one. The power-up process is determined by the manufacturing process deviation. Due to unpredictable and uncontrollable manufacturing process deviation, the power-on value of the cell is unique after manufacturing and can be considered as a type of SRAM fingerprint.

**Figure 2:**
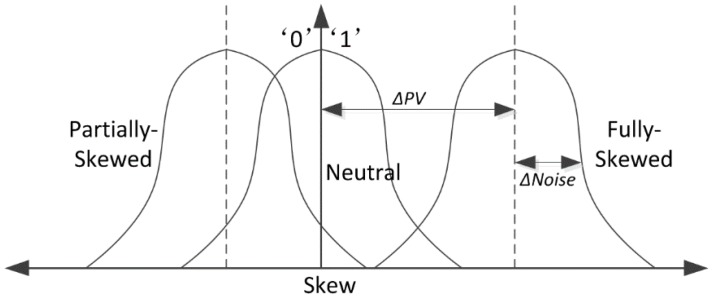
Skew feature of SRAM cells [8].

The power-on value of a 6T SRAM cell can be measured by the skew as shown in Figure 2. The area covered by the intersection of the curve and the vertical axis indicates the trend of the cell’s power-on value. The bit-1 cell has a large area on the one side than zero side, and vice versa for a bit-0 cell. Δ*PV* is the manufacturing process and voltage deviation, and Δ*Noise* is the noise deviation. When the Δ*PV* and Δ*Noise* of a cell are larger, the area difference is greater, and the corresponding skew is larger. As a result, the cell presents a strong one or strong zero attribute.

### 2.2. Structure of SRAM PUFs

**Figure 3:**
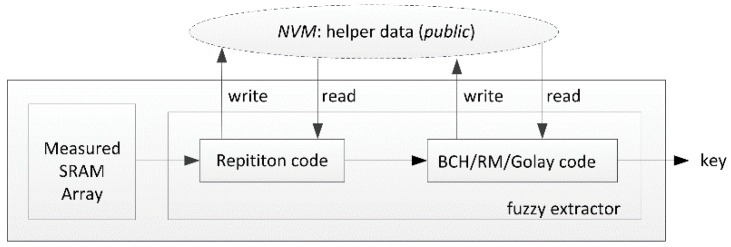
Structure of a SRAM PUF.

The power-on value of a 6T SRAM cell is basically determined by manufacturing process deviation [24], but it is also disturbed by other environmental factors, such as temperature and the ramp-up time of the power supply [25]. This means the power-on value has slight noise. To ensure the key can be correctly extracted from the SRAM PUF each time, a fuzzy extractor is needed to process the noisy SRAM responses, correct the noise by using the helper data generated in the enrollment step, and then, recover the key. Figure 3 shows the basic structure of SRAM PUFs. Error correction code in fuzzy extractors is usually concatenated with two codes. The repetition code $C_{rep}\left(n,k,t\right)$ [16], where *n* is the length of the input data, *k* is the dimension of the input data and *t* is the number of errors it can correct, can be implemented with only 2×(n−1) exclusive OR (XOR) gates. Therefore, repetition code is very efficient for use in PUFs as one of the error correction codes.

There are two phases in PUF applications: the enrollment phase and reconstruction phase. The helper data, which is generated in the enrollment phase, is used to recover the secret key in the reconstruction phase. The functions of repetition code in two phases are as follows:

*Enrollment phase*: Challenge the SRAM array, and measure the response *R*, and calculate the helper data H=R mod G, where *G* is the generator polynomial. The helper data is stored in public non-volatile memory and can be accessed by hardware and/or software.

*Reconstruction phase*: Challenge the SRAM array, and measure the response $R^{\prime }$, perform error correction process to obtain $R_{ecc}=\left(R^{\prime }\;mod\;G\right)⊕H=E_{rr}\;mod\;G$, where $E_{rr}$ is the error vector, recover $E_{rr}$ from $R_{ecc}$, and then calculate the corrected $R^{\prime \prime }=E_{rr}⊕R^{\prime }$. The error-corrected response $R^{\prime \prime }$ is ideally equal to *R*.

### 2.3. Entropy of SRAM PUFs

Entropy is a measure of the uncertainty about the outcome of an observation of x. Closely related to Shannon entropy is min-entropy $H_{\infty }\left(x\right)$, Min-entropy provides a lower bound of the amount of entropy contained within a random process by only taking the most likely outcome into account. $p_{0}$ and $p_{1}$ are the probability of 0 or 1 respectively, and $p_{0}+p_{1}=1$. Min-entropy [16] is:

(1) $$H_{\infty }\left(x\right)=-log_{2}\left[max\left(p_{0},p_{1}\right)\right]$$

Consider n-bit PUFs responses and a cycling error correcting code C(n,k,t). After error correcting, the leftover entropy is:

(2) $$H_{left}\left(x\right)=k-\left(1-H_{\infty }\left(x\right)\right)\times n$$

## 3. Cloning SRAM Power-on Value

### 3.1. Weakness of SRAM PUFs Based on a Repetition Code

For the repetition code $C_{rep}\left(n,k,t\right)$, the generation polynomial G is generally $G=\sum_{i=0}^{n-1}x^{i}$ [16]. Taking *n*-bit responses $R=\sum_{i=0}^{n-1}y_{i}$ as input, (*n* − 1)-bit helper data $H=\sum_{i=0}^{n-2}h_{i}$ is generated, where $h_{i}=y_{0}⊕y_{i+1}$. There is no helper data generated in position $y_{0}$, and we can obtain $h_{0}$ from position $y_{1}$, $h_{1}$ from position $y_{2}$ and so on.

**Figure 4:**
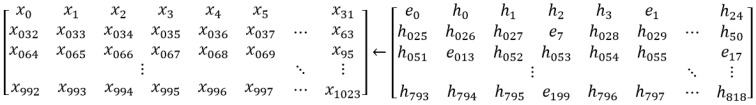
Mapping between the clone matrix *E* and response *R*.

Due to the linear nature of repetition codes, a certain relationship exists between the helper data and the response *R*. Figure 4 shows the relationship between response *R* of a 32 × 32 SRAM array and the generated helper data with repetition code $C_{rep}\left(5,1,2\right)$. The SRAM array has 32 columns and 32 rows, and their power-on values are listed in the left matrix one-by-one as $x_{0}$, $x_{1}$, ⋯ $x_{1023}$. In enrollment phase, the power-on values are measured as response *R* and every five elements are sent to $C_{rep}\left(5,1,2\right)$ to obtain 4-bit helper data, and we obtain a total of 819-bit helper data, listed in the right matrix as $h_{0}$, $h_{1}$, ⋯, $h_{818}$. An empty element $e_{z}$ where z∈(0,n−1) with initial value zero is inserted at position $y_{0}$ where helper data is not generated. The right matrix composed by helper data and $e_{z}$ creates an initial clone matrix *E*:

The generation formula of the helper data shows that when $y_{0}=0$, $h_{i}=y_{i+1}$, that is, the corresponding response *R* and the helper data are the same under this condition. The matrix *E* is the same as the response *R* when $y_{0}=0$, and the matrix *E* is the opposite of response *R* when $y_{0}=1$.

From the analysis above, whether the matrix *E* is the same as the response *R* is determined by the hiding $y_{0}$. In the worst case, the probability of the equality between *E* and response *R* is $1/2^{\frac{m}{n}}$, where *m* is the length of response *R* and *n* is the length of the input data of the selected repetition code. $q_{y0}$ is the ratio of having 0 in responses, i.e., $q_{y0}=n_{yo}/m$, $n_{yo}$ is the number of cells with a power-on value of 0. When $q_{y0}$ is higher, the probability of having $y_{0}=0$ is also higher. The similarity between the matrix *E* and response *R* is higher too, and *E* directly leaks most of the information about response *R*.

For repetition code $C_{rep}\left(n,k,t\right)$, we can obtain one dimension code $C_{rep}\left(n-k+1,1,t\right)$ by shortening the dimension by k−1 bits. The two codes have the same generator polynomial and error correction capabilities [16], usually a code $C_{rep}\left(n,1,t\right)$ is chosen for fuzzy extractor. According to Equation (2), only 1-bit entropy is left after error correction by repetition code, and the left entropy bit is hiding $y_{0}$. Ideally, $p_{0}$ and $p_{1}$ in position $y_{0}$ have same value of 50%, but in the real world, the value may be biased to 0 due to manufacturing process variations and changes in power-on environment (temperature, voltage, etc.). $q_{y0}$ can be considered as the observed value of $p_{0}$ in position $y_{0}$. When this bias is heavy, the leftover entropy will decrease to zero. For repetition code $C_{rep}\left(5,1,2\right)$, leftover entropy will be zero when $q_{y0}>57.4\%$ according to Equations (1) and (2). The responses with high $q_{y0}$ are considered weak responses.

### 3.2. Effect of Strongest Cell Distribution in a SRAM Array on PUF Security

The difference between the matrix *E* and PUFs’ response *R* is determined by the first bit $y_{0}$. Using the exhaustive method, traversing $y_{0}$ for every *n*-bit data requires considerable time and effort to obtain the correct *E*. However, with the distribution of the strongest cells in the SRAM array, the data in matrix *E* can be partially corrected to reduce the difference between the matrix *E* and the response *R*.

The correlation between cell *S*(*i*,*j*) and its closest cells is higher than with cells farther away: i is the row number of SRAM array and j is the column number. The correlation decreases with increasing distance. It is highly probably that a strongest cell is surrounded by cells with the same power-on value. According to this feature, we designed the following recovery algorithm.

The enrollment procedure is performed *N* times to obtain *N* sets of helper data, denoted as $H_{1}\left(x\right),H_{2}\left(x\right),H_{3}\left(x\right),\ldots ,H_{N}\left(x\right)$, where x∈[0, l−1], l is the length of the helper data. For $C_{rep}\left(n,k,t\right)$, each group of helper data consists of l/(n−1) blocks marked as $B_{c}\left(0\right),B_{c}\left(1\right),\ldots ,B_{c}\left(l/\left(n-1\right)-1\right)$, where c is the set number or helperdata and *c* ∈[0,N]. To describe simplicity, the first set of response *R* is chosen as the response to be cloned. From the analysis above, when the error bit occurs in the $y_{0}$ position, the generated helper data of each set are opposite to each other. Therefore, the helper data need to be pre-processed one–by-one according to the block length (n−1) so the helper data truly reflects the power-on trend.

First, calculate the difference of the two helper data block-by-block:

(3) $$Diff=B_{1}\left(f\right)⊕B_{c}\left(f\right)$$

where f is the block number and f∈[0, l/(n−1)−1]. Then, we recover the block according to *Diff*:

(4) $$B_{c}^{\prime }\left(f\right)=\{\begin{array}{c} B_{c}\left(f\right),Diff\;\leq t\\ \bar{B_{c}\left(f\right)},Diff\;>t \end{array}$$

where *t* is the error correction capability of the repetition code. The error-corrected helper data are $H_{c}^{\prime }\left(x\right)=\sum_{f=0}^{l/\left(n-1\right)-1}B_{c}^{\prime }\left(f\right)$. The weighted sum of the cell in *N* helper data at location *x* is:

(5) $$P=\frac{\sum_{c=1}^{N}H_{c}^{\prime }\left(x\right)}{N}$$

A cell is a strong cell if *P* is close to 1 or 0. However, we cannot confirm whether it is a strong-1 cell or a strong-0 cell. We use $T_{\mathrm{th}}$ as the decision threshold for strong cells and $T_{\mathrm{th}}\in \left[0,\;100\%\right]$, i.e., when $P\leq \left(1-T_{\mathrm{th}}\right)$ or $P>T_{\mathrm{th}}$, we label it as strong cell, otherwise it is a metastable cell. By analyzing the *P* value in the corresponding position of the helper data in matrix *E*, we can identify whether the SRAM array corresponding to position *x* is a strong cell. Through the above analysis and processing, an element in matrix *E* has two attributes: one for its value, 0 or 1, and the other for the property of the cell, that is, whether it is a strong cell. Empty elements have initial attributes of value 0 and non-strong cell.

The strongest cell in the SRAM is surrounded by cells with the same power-on tendency. When a series of cells in a group of helper data have the same trend of power-on value, the cell in the middle position is taken as strongest cell S(i,j). The correlation of the surrounding cells, $S_{area}$, is generated by separately calculating the weight.

**Figure 5:**
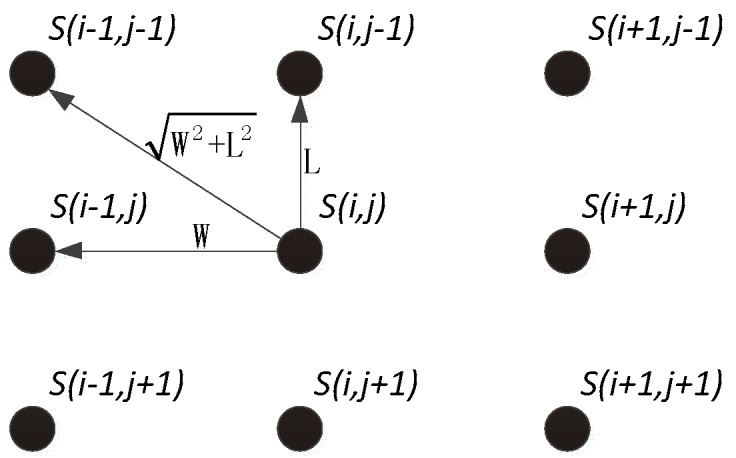
Structure of a 3 × 3 cell array in two-dimension space.

As shown in Figure 5, the position S(i,j) to be calculated is a known strongest bit. There are eight cells, S(i−1,j−1),S(i,j−1),S(i+1,j−1),S(i−1,j),S(i+1,j),S(i−1,j+1), S(i,j+1) and S(i+1,j+1), around the cell S(i,j). The correlation in this area is calculated as:

(6) $$\begin{array}{cc} S_{area}=\frac{1}{8}P_{S\left(i-1,j-1\right)} & +\frac{\sqrt{W^{2}+L^{2}}}{8L}P_{S\left(i,j-1\right)}+\frac{1}{8}P_{S\left(i+1,j-1\right)}+\frac{\sqrt{W^{2}+L^{2}}}{8W}P_{S\left(i-1,j\right)}\\ & +\frac{\sqrt{W^{2}+L^{2}}}{8W}P_{S\left(i+1,j\right)}+\frac{1}{8}P_{S\left(i-1,j+1\right)}+\frac{\sqrt{W^{2}+L^{2}}}{8L}P_{S\left(i,j+1\right)}\\ & +\frac{1}{8}P_{S\left(i+1,j+1\right)} \end{array}$$

where *W* is the width of the SRAM cell and *L* is the length as shown in Figure 1. If there are empty elements in the eight surrounding cells, then the weighted sum of the corresponding empty element is calculated as follows:

(7) $$P=\{\begin{array}{c} 0,e_{z}=0\\ 1,e_{z}=1 \end{array}$$

If $S_{area}$ has the same tendency as $P_{S\left(i,j\right)}$, note the value of $y_{0}$ corresponding to each cell at this time, otherwise adjust the value of $y_{0}$ corresponding to the neighbor cell S(i,j) or itself. The values of the neighboring cells are updated synchronously, and the weighted sum corresponding to the cell is updated to 1−P. Then update the matrix *E* and re-calculate $S_{area}$.

The updated clone matrix is label as *E’*. After the above method is processed, the elements in *E’* are transformed as follows:

(8) $$k_{i,j}=\{\begin{array}{c} 0,E^{\prime }\left(i,j\right)\leq \left(1-T_{\mathrm{th}}\right)\\ 1,E^{\prime }\left(i,j\right)>T_{\mathrm{th}}\; \end{array}$$

The new matrix $K=\sum k_{i,j}$ is considered as the final clone matrix of response *R*.

## 4. Proposed New Fuzzy Extractor

To solve the problem of information leakage of the strongest cells, we propose an Error Resistant Fuzzy Extractor (ERFE), which extracts the value of the sum of multiple cells as output key.

### 4.1. ERFE Architecture

The value of sum of 11-bit cells is:

(9) $$sum\left(11\right)=\sum_{o=0}^{10}x_{o}$$

As explained in the previous section, the power-on value of a cell is almost determined after manufacturing, and can be extracted as a key. After the manufacturing process, sum(11) is stable too. Due to the linear relationship between the power-on value and the sum value, sum(11) can be regarded as another type of fingerprint for SRAM. We propose a novel extraction scheme, ERFE, which allows 4-bit errors. ERFE is still divided into two phases: enrollment and reconstruction. In the enrollment phase, *Mask* and *AddOp* signals will be generated.

In Algorithm 1, *AddOp* indicates whether the addition or subtraction operation is performed in the reconstruction phase, and the operand of addition or subtraction is “1”. *Mask* indicates whether there 11-bit data are valid. When *Mask* equals 1, 11-bit data are invalid and cannot be used for key extraction. When *AddOp* and *Mask* are produced in the enrollment phase, they are also stored in non-volatile memory, which can also be considered a type of helper data.

**Algorithm 1.** Generation algorithm of *Mask* and *AddOp***Input:** 11-bit responses of SRAM $x_{0}$, $x_{1}$, $x_{2}$, …, $x_{10}$ **Output:**
*Mask, AddOp* 1.    initialize *Mask*, *AddOp* with zero 2.    $sum=x_{0}$ + $x_{1}+x_{2}+$…+ $x_{10}$ 3.    **if**
(sum==0) **then** 4.    *AddOp* = 1; *Mask* =0; 5.    **else if**
(sum==2) **then** 6.    *AddOp* =0; *Mask* = 0; 7.    **else if**
(sum==9) **then** 8.    *AddOp* = 1; *Mask* = 0; 9.    **else if**
(sum==11) **then** 10.   *AddOp* = 0; *Mask* = 0; 11.   **else then** 12.   *AddOp* = 0; *Mask* = 1; 13.   **end if** 14.   **Return**
*Mask, AddOp*

In the reconstruction phase, the responses $R^{\prime }$ are divided to multiple blocks, every block contains 11-bit data. For each block, when *Mask* equals 0, this block is valid, and the $sum^{\prime }$ is calculated using Equation (9), then we obtain $sum^{\prime \prime }$ using *AddOp*:

(10) $$sum^{\prime \prime }=\{\begin{array}{c} sum^{\prime }+1,\;\mathrm{AddOp}==1\\ sum^{\prime }-1,\;\mathrm{AddOp}==0 \end{array}$$

Equation (10) contains an addition and subtraction operation. Overflow or underflow may occur when bit error happens. When performing an addition operation and overflow occurs, let $sum^{\prime \prime }$ = 11, and $sum^{\prime \prime }$ = 0 when underflow occurs. Since we have $sum^{\prime \prime }$, we can recover the output key:

(11) $$\mathrm{Key}=\{\begin{array}{c} 0,\;sum^{\prime \prime }\leq 5\\ 1,\;sum^{\prime \prime }>5 \end{array}$$

### 4.2. ERFE Security Analysis

**Figure 6:**
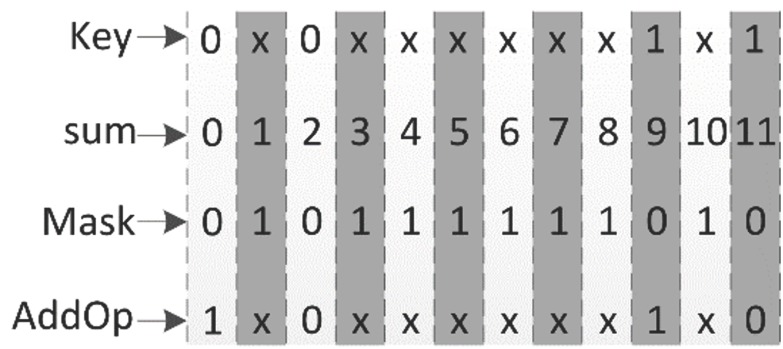
Diagram of *AddOp*, *Mask*, *sum* and *Key*.

The main target of the PUF is security and when using this fuzzy extractor, the key clearly cannot be obtained from the helper data, which is accessible to everyone. Figure 6 shows the corresponding diagrams of *AddOp*, *Mask*, *sum*, and *Key* in the enrollment phase. “-” means this area is invalid:

From Algorithm 1 and Equations (10) and (11), *AddOp* and the final key have a certain overlap. In Figure 6, *sum* = 2 and *sum* = 9 overlap with *AddOp* and *Key*, *AddOp* and *Key* are equal within this overlap interval, and $r_{0}$ is the accurate rate that *AddOp* is regarded as *Key*:

(12) $$r_{0}=\frac{p_{sum=2}+p_{sum=9}}{\left(p_{sum=0}+p_{sum=2}+p_{sum=9}+p_{sum=11}\right)}$$

The rate of using the opposite value of *AddOp* as the key is $r_{1}=1-r_{0}$. $P_{Key}$ is the rate of guessing *Key* from *AddOp* and $P_{Key}=max\left(r_{0},r_{1}\right)$. $P_{Key}$ was ideally expected to close to 50% when the sum value is uniformly distributed. The sum value was not uniformly distributed because of the production skew is unpredicted and uncontrollable, so the output of the corresponding *Key* also has some bias.

### 4.3. Stability Analysis of ERFE

The probability that a string of *n* bits has more than *s bits* errors is calculated as [26]:

(13) $$p_{s}=\overset{n}{\underset{i=s+1}{\sum }}C_{n}^{i}\;p_{b}^{i}\;{\left(1-p_{b}\right)}^{n-i}$$

where $p_{b}$ is the bit error rate. In Figure 6, when sum∈(0,2,9,11), *Mask* equals 0, so 11-bit data is used to extract *Key*. The number of errors in each group of 11-bit data that changes from 0 to 1 is recorded as $N_{0\to 1}$, and the number of errors changing from 1 to 0 is recorded as $N_{1\to 0}$. As shown in Equations (10) and (11), we obtain *Key* = 0 and *sum* = 0 or 2 in the enrollment phase. In the reconstruction phase, we obtain $sum^{\prime \prime }=sum+(N_{0\to 1}-N_{1\to 0})\pm 1$, *Key* extraction will return a wrong value if $N_{0\to 1}-N_{1\to 0}\geq 5$. Similarly, key extraction will return a wrong value if $N_{1\to 0}-N_{0\to 1}\geq 5$ at *sum* = 9 or *sum* = 10. ERFE can accommodate 4-bit errors in 11-bit data. If more than 4-bit errors occur ERFE does not work correctly when it meets the condition of $\left|N_{0\to 1}-N_{1\to 0}\right|\geq 5$.

p(n,e) is the probability that has e bit errors in n bits string, and $N_{1\to 0}+N_{0\to 1}=e$. p(e,5) is the probability of $\left|N_{0\to 1}-N_{1\to 0}\right|\geq 5$, assuming an error event that changes from 0→1 or 1→0 occurs with the same probability. The rate of extracting a wrong value is:

(14) $$P_{FE}=\overset{n}{\underset{e=5}{\sum }}p\left(n,e\right)p\left(e,5\right)=\overset{n}{\underset{e=5}{\sum }}(C_{n}^{e}\;p_{2}^{e}\;{\left(1-p_{2}\right)}^{n-e}\;\frac{\sum_{i=e/6+5}^{e}C_{e}^{i}}{2^{e}})$$

In Equation (14), $p_{2}=p_{s}$ if an error correcting code (ECC) like repetition code or BCH code is concatenated [14], otherwise, $p_{2}=p_{b}$. γ is the length of extracted key, and the ERFE failure rate is:

(15) $$P_{fail}=1-{\left(1-P_{FE}\right)}^{\gamma }$$

Table 1 gives the result of $P_{fail}$ with different $p_{2}$. To obtain a failure rate less then $10^{-6}$. ERFE needs to use the SRAM cell with $p_{2}$ < 0.01. A method to find the stable bit with lower $p_{b}$ was reported [21], and a stable bit rate of 59.26% in 6KB SRAM was achieved after power-voltage variation, temperature variation and aging effects tests. They also proposed Neighborhood-Base Bit Selection Algorithm to determine the stable bits.

**Figure 7:**
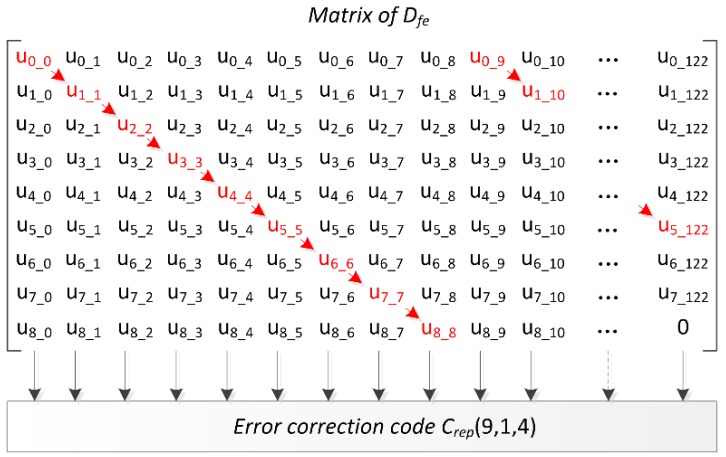
Diagrams of $D_{fe}$.

Another method of obtaining lower $P_{fail}$ is to add the error correction code after the SRAM responses are measured. The data are chosen by *Mask*, and invalid data are discarded. Since not all the SRAM cells are used and the mapping relationship of Figure 4 is broken, repetition code will not leak the information of the strongest cells. Repetition code $C_{rep}\left(9,1,4\right)$ is used in ERFE. All the valid blocks create a matrix $D_{fe}$ with nine rows. Each column of 9-bit data is sent to repetition code separately, and the first bit $y_{0}$ in repetition code is chosen in different row. Figure 7 provides an example of $D_{fe}$ with 100 blocks, so we obtain 1100 elements, the $D_{fe}$ is a matrix with 9 rows and 123 columns, and $u_{a\_b}$ is the element of $D_{fe}$ which means it is at the *a*-th row and *b*-th column, *a* ∈(0, 8), *b* ∈(0, 122). The last 123×9−1100=7 empty elements are filled to zero. Each column data are sent to repetition code one at a time, and the data shown in red are considered as $y_{0}$ in each column. ERFE was designed symmetrically. The opposite value of *sum* = 0 and *sum* = 2 is *sum* = 11 and *sum* = 9, so the opposite value of the key is generated. The probability of guessing the sum value using helper data generated by repetition code is 50%.

.

## 5. Experiment and Analysis

### 5.1. Experimental Set-Up

**Figure 8:**
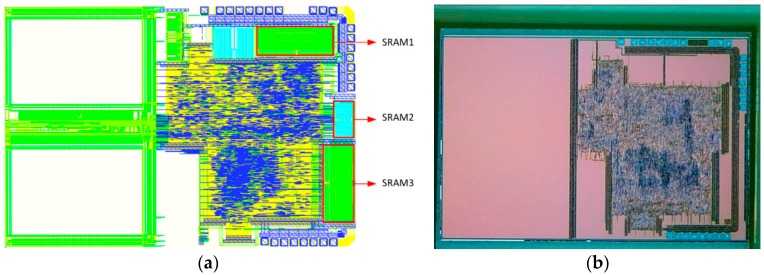
The layout and photograph of test chip (**a**) layout and (**b**) photograph.

Our experimental data were generated from System on Chip (SoC) chips that are manufactured in a HHGRACE 130 nm CMOS technology multi-project wafer run. These chips have three independent SRAM arrays as shown in Figure 8. The size of the SRAM1 and SRAM3 were 2048 × 32, whereas the size of the SRAM2 was 512 × 32. These SRAM blocks are standard components provided by the foundry, compiled by the memory compiler. These SRAMs are powered by the low dropout regulator (LDO) of the ASIC. The power supply range of the chip is 1.65 to 5.5 V. The LDO output is 1.62 to 1.98 V. The max supply voltage drop of these SRAMs is 30 mV.

**Figure 9:**
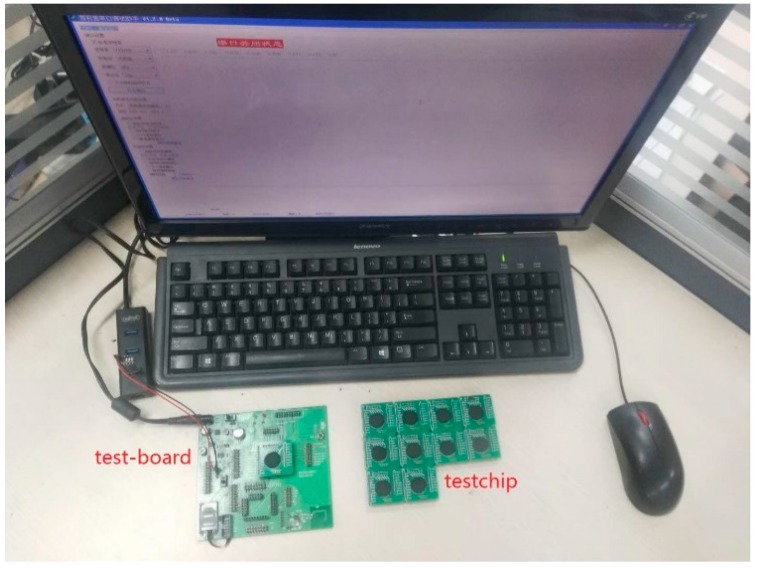
Experimental platform for verification of the proposed methods.

These three SRAMs were used to generate CRPs, and we used the last 32 × 32 blocks for experiments. The experimental platform, as shown in Figure 9, included the following equipment:

(1) A desktop computer responsible for data receipt and analysis, and (2) a test-board with a USB-to-RS232 cable responsible for transmitting the power-on value of the test chip.

When the power is on, the test-board sends three SRAMs’ power-on values to the desktop. After the transmission is over, turn the power off, and wait for more than five minutes to turn power on. Next, perform the transmitting again until all 100 samples are generated. The five-minute waiting time between two transmissions ensures that there is no residual charge affecting the power-on value. The test temperature was 25 °C. After all samples are transmitted, they are processed and analyzed on a desktop. $C_{rep}\left(5,1,2\right)$ was chosen for the cloning experiment.

### 5.2. Characteristics of the Power-on Value of SRAMs

Intra distance is the difference between two responses of one SRAM, and the maximum intra distance can be considered as an observed value of $p_{b}$. Inter distance is the difference between two samples of two different SRAM. Table 2 shows two distances of the tested SRAMs.

The weighted sum P is calculated by 100 response samples according to Equation (5), and Table 3 shows the distribution. The corresponding metastable bits with 0<P<1 are 34.18, 28.13 and 14.26% respectively. The stable-0 bits with P = 0 are 34.18%, 40.72% and 44.14%, respectively. The stable-0 bits in the three SRAMs are slightly higher than stable-1 bits.

### 5.3. Weak Responses

**Figure 10:**
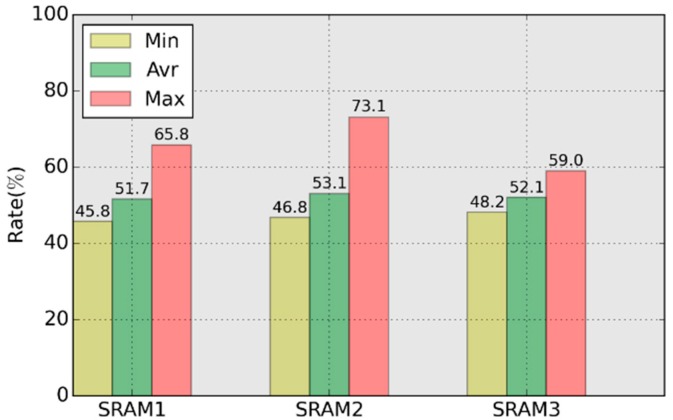
$q_{y0}$ for the tested SRAMs.

Figure 10 shows the maximum, average and minimum value of $q_{y0}$ in 100 responses. The maximum value for SRAMs are 65.8%, 73.1% and 59.0% respectively. Using the response with maximum $q_{y0}$ will lead to zero leftover entropy in the reconstruction phase according to Equation (2).

Table 4 shows the distribution of $q_{y0}$ in 100 samples. A response with $q_{y0}$ > 57.4% is considered weak response, and the distribution of weak responses in SRAMs is 7.8%, 6%, 1.2% respectively. From the statistical results, the weak responses appear randomly, and the probability of its occurrence is large.

.

### 5.4. Leakage of Strongest Cells

The samples of the responses are sent into the PUF, and the enrollment operation is performed 100 times to obtain 100 sets of helper data, which are denoted as $H_{1}\left(x\right),H_{2}\left(x\right),H_{3}\left(x\right)\ldots H_{100}\left(x\right)$. Choosing response with maximum $q_{y0}$ as response to be cloned, and the helper data ($H_{1}\left(x\right)$) generated by the chosen response and the empty cells $e_{z}$ consist of a clone matrix *E*.

The *Diff* is sequentially calculated according to Equation (3). The parameter *t* is two corresponding to $C_{rep}\left(5,1,2\right)$. Then $H_{i}^{\prime }\left(x\right)$ is obtained according to Equation (4). Table 5 shows the distribution of the weighted sum percentage $P_{h}$ of each helper data calculated according to Equation (5). Due to the errors in the helper data adjustment in Equations (3) and (4), the ratio of $P_{h}$ = 0 and $P_{h}$ = 1 are reduced compared to P shown in Table 3. In this experiment, let $T_{\mathrm{th}}$ = 0.9, meaning a cell with $P_{h}$ < 0.1 and $P_{h}\;$> 0.9 is a strong cell.

If all of the four helper data have same power-on tendency of zero, choose the second one as strongest cell *S*(*i*,*j*). According to Equation (6), the value of $e_{z}$ corresponding to the strongest cell *S*(*i*,*j*) or the surrounding cells around is adjusted. The elements in the clone matrix *E* are sequentially processed, and a final clone matrix *K* is obtained according to Equation (8). Table 6 shows the similarity of the response *R* and the clone matrix, where $N_{cells}$ is the number of strongest cells, $\delta_{1}$ is the similarity of the response *R* and initial clone matrix *E*, $\delta_{2}$ is the similarity of response *R* and the clone matrix *K* corrected using the distribution of strongest cells, and Δδ is the repair rate obtained by leakage of strongest cells.

Based on the clone matrix obtained from the weak response, the similarity increased by 8.3%, 5.9% and 7.7% respectively. Moreover, the greater the strongest-cells exist in the SRAM array, the greater the similarity increase.

Notably, the above repair process can be performed only when the cloned matrix and SRAM array meet the relationship shown in Figure 4. The strongest cells distribution characteristics also fully or partially appears in matrix *E*.

### 5.5. Results of ERFE

**Figure 11:**
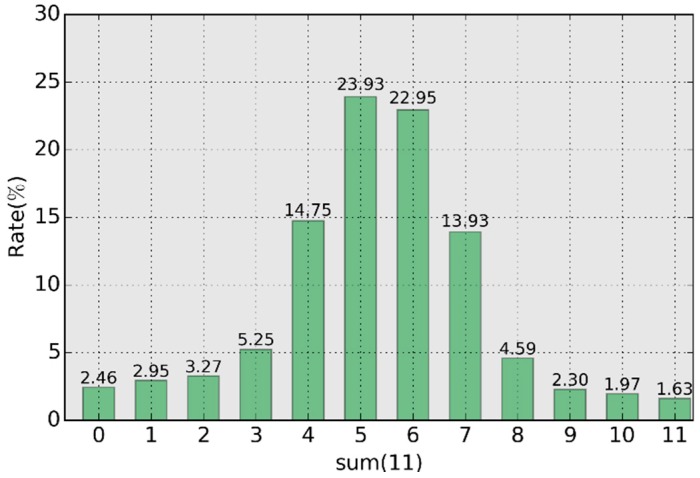
Distribution of the sum value.

For the data in SRAM1, Figure 11 provides the statistical distribution of the sum value according to Equation (9). The sum value is relatively concentrated in the region of (4,5,6,7), reaching 75.56%. The distribution in the region (0,2,9,11) is low, only 9.66%, meaning only 9.66% cells are valid for use in ERFE. The probability was determined to be $P_{key}=57.66$% according to Equation (12).

Table 7 compares ERFE and other Hard FE. $N_{help}$ is the size of the helper data, and $N_{\mathrm{Mask}}$ and $N_{\mathrm{AddOp}}$ are the number of *Mask* and *AddOp* respectively. $ERFE_{\mathrm{ecc}}$ is the ERFE with error correction code, and $ERFE_{\mathrm{stable}}$ is the ERFE with stable bits. $p_{b}$ in $ERFE_{\mathrm{stable}}$ is 0.01, and the others are 0.15. Min-entropy of all FEs are 95%, a total of 128-bit keys are extracted, entropy loss in randomness extraction is 128-bit, γ=256, and *BCH* is BCH code.

We provide the results of $ERFE_{\mathrm{stable}}$ obtained using the method proposed in Xiao et al. [21]: 59.26% of the cells are stable bit and can be used as the input for ERFE. $N_{puf}=256\times 11÷0.0966÷0.5926=49,126$, $N_{help}=N_{\mathrm{Mask}}+N_{\mathrm{AddOp}}=49,126÷11+256=4722$, and $P_{fail}=3.5\times 10_{-7}$. We also obtained a result of $P_{fail}$ as low as 1.8 × 10^−8^ with an error correcting code $C_{rep}\left(9,1,4\right)$, and $p_{b}=0.15$. In this case, $N_{puf}=256\times 11÷0.0966=29,161$, $N_{help}=N_{\mathrm{Mask}}+N_{\mathrm{AddOp}}+N_{\mathrm{rep}}=29,161÷11+256+256\times 11÷9\times 8=5410$, and $N_{\mathrm{rep}}$ is the size of helper data generated by $C_{rep}\left(9,1,4\right)$. Because only 9.66% of cells are valid, ERFE needs a larger $N_{puf}$ than other Hard FEs.

We synthesized and mapped ERFE in the Xilinx Spartan-3E-500 FPGA platform. Bösch et al. [14] provided the FPGA resources of $C_{rep}\left(3,1,1\right)$, $C_{rep}\left(5,1,2\right)$, $C_{rep}\left(7,1,3\right)$, and $C_{rep}\left(9,1,4\right)$ using 41 slices. ERFE almost requires the same slice resources as repetition code as shown in Table 7. However, the BCH algorithms themselves are much more complex: thus, that their hardware complexity is expected to be similarly higher. We estimate that ERFE is much more efficient than Hard FE in terms of hardware resource requirements. Our proposed ERFE is space efficient and suitable for use in resources-limited device such as Radio Frequency Identification (RFID) and IoT and so on.

## 6. Conclusions

In this paper, we analyzed the physical challenge space of SRAM PUFs, and found a weak response in the SRAM PUF based on a repetition code. The presence of strongest cells in SRAM can cause public helper data generated by a repetition code to leak more information about the responses. Using the helper data generated by the weak responses, the leftover entropy of PUF was zero. Our research experimentally confirmed that the pitfall of using repetition codes [15] exists, and the distribution of strongest cells causes helper data to reveal more information about PUFs’ responses, which further decreases the leftover entropy of the PUF based on repetition codes. We proposed ERFE which does not leak information of the strongest cells. ERFE uses cells’ sum value as the key with 4-bit error tolerance ability. ERFE is as lightweight as a repetition code, and is also very suitable for implementation in FPGA or software. Future work will include reducing the size of $N_{puf}$, and testing ERFE with additional types of SRAM chips and other types of PUFs.

## Figures and Tables

**Table 1:** $P_{fail}$ with different $p_{2}$ (γ=256).

$p_{2}$	0.15	0.1	0.05	0.02	0.01
$P_{fail}$	0.12	0.022	9.8 × 10^−4^	1.1 × 10^−5^	3.5 × 10^−7^

**Table 2:** Intra distance and inter distance of test SRAMs.

Scheme	SRAM1		SRAM2		SRAM3	
	Min	Max	Min	Max	Min	Max
Intra distance	0.5%	11.7%	0.2%	13.4%	0.4%	7%
Inter distance	44%	53%	48%	50%	49%	52.5%

**Table 3:** Distribution of the weighted sum of responses.

*P*	SRAM1	SRAM2	SRAM3
0	34.18%	40.72%	44.14%
0 < P < 0.1	8.98%	4.30%	3.42%
0.1 < P < 0.3	1.56%	1.56%	1.27%
0.3 < P < 0.7	3.91%	4.79%	4.10%
0.7 < P < 0.9	2.34%	0.98%	1.27%
0.9 < P < 1	17.38%	16.50%	4.20%
1	31.64%	31.15%	41.60%

**Table 4:** Distribution of $q_{y0}$.

*q*_*y*0_	SRAM1	SRAM2	SRAM3
$q_{y0}$ > 70%	0	6%	0
57.4% < $q_{y0}\;$≤ 70%	7.8%	0	1.2%
50% < $q_{y0}\;$≤ 57.4%	56.2%	71%	79.8%
40% < $q_{y0}\;$≤ 50%	36%	23%	19%

**Table 5:** Distribution of the weighted sum of the helper data.

*P_h_*	SRAM1	SRAM2	SRAM3
0	16.54%	19.61%	19.49%
0 <$\;P_{h}\;$< 0.1	30.15%	26.59%	29.53%
0.1 <$\;P_{h}\;$< 0.3	3.92%	3.43%	4.17%
0.3 <$\;P_{h}\;$< 0.7	8.58%	8.7%	8.82%
0.7 <$\;P_{h}\;$< 0.9	4.78%	1.96%	1.35%
0.9 <$\;P_{h\;}$< 1	25.86%	30.39%	22.06%
1	10.17%	9.32%	14.58%

**Table 6:** Comparison of the clone matrix and response *R*.

	SRAM1	SRAM2	SRAM3
$N_{cells}$	23	14	22
$\delta_{1}$	65.8%	73.1%	59.0%
$\delta_{2}$	74.1%	79.0%	66.7%
Δδ	8.3%	5.9%	7.7%

**Table 7:** Result of different fuzzy extractors (FEs).

Scheme	*C*_1_(n_1_,k_1_,t_1_)	*C*_2_(n_2_,k_2_,t_2_)	$l_{2}$	$N_{puf}$	$N_{help}$	$P_{fail}$	Slice
Hard FE [15]	$C_{rep}\left(3,1,1\right)$	*BCH*(977,232,102)	3	8793	8097	1.9 × 10^−7^	41 [14]
Hard FE [15]	$C_{rep}\left(5,1,2\right)$	*BCH*(982,502,53)	1	4910	4408	8.2 × 10^−7^	41 [14]
Hard FE [15]	$C_{rep}\left(7,1,3\right)$	*BCH*(817,542,28)	1	5719	5177	4.9 × 10^−7^	41 [14]
$ERFE_{\mathrm{ecc}}$	$C_{rep}\left(9,1,4\right)$	-	-	29,161	5410	1.8 × 10^−8^	80
$ERFE_{\mathrm{stable}}$	-	-	-	49,126	4722	3.5 × 10^−7^	39
